# Correlation between short- and mid-term hemoglobin A1c and glycemic control determined by continuous glucose monitoring

**DOI:** 10.1186/s13098-021-00714-8

**Published:** 2021-09-06

**Authors:** Jen-Hung Huang, Yung-Kuo Lin, Ting-Wei Lee, Han-Wen Liu, Yu-Mei Chien, Yu-Chun Hsueh, Ting-I Lee, Yi-Jen Chen

**Affiliations:** 1 Division of Cardiovascular Medicine, Department of Internal Medicine, Wan Fang Hospital, Taipei Medical University, Taipei, Taiwan; 2 Division of Cardiology, Department of Internal Medicine, School of Medicine, College of Medicine, Taipei Medical University, Taipei, Taiwan; 3 Cardiovascular Research Center, Wan Fang Hospital, Taipei Medical University, Taipei, Taiwan; 4 Division of Endocrinology and Metabolism, Department of Internal Medicine, School of Medicine, College of Medicine, Taipei Medical University, 111 Xinglong Road, Section 3, Wenshan District, Taipei, 11696 Taiwan; 5 Division of Endocrinology and Metabolism, Department of Internal Medicine, Wan Fang Hospital, Taipei Medical University, Taipei, Taiwan; 6 Department of General Medicine, School of Medicine, College of Medicine, Taipei Medical University, Taipei, Taiwan; 7 Graduate Institute of Clinical Medicine, Taipei Medical University, Taipei, Taiwan

**Keywords:** Diabetes mellitus, Continuous glucose monitoring, Hemoglobin A1c

## Abstract

**Background:**

Glucose monitoring is vital for glycemic control in patients with diabetes mellitus (DM). Continuous glucose monitoring (CGM) measures whole-day glucose levels. Hemoglobin A1c (HbA1c) is a vital outcome predictor in patients with DM.

**Methods:**

This study investigated the relationship between HbA1c and CGM, which remained unclear hitherto. Data of patients with DM (n = 91) who received CGM and HbA1c testing (1–3 months before and after CGM) were retrospectively analyzed. Diurnal and nocturnal glucose, highest CGM data (10%, 25%, and 50%), mean amplitude of glycemic excursions (MAGE), percent coefficient of variation (%CV), and continuous overlapping net glycemic action were compared with HbA1c values before and after CGM.

**Results:**

The CGM results were significantly correlated with HbA1c values measured 1 (r = 0.69) and 2 (r = 0.39) months after CGM and 1 month (r = 0.35) before CGM. However, glucose levels recorded in CGM did not correlate with the HbA1c values 3 months after and 2–3 months before CGM. MAGE and %CV were strongly correlated with HbA1c values 1 and 2 months after CGM, respectively. Diurnal blood glucose levels were significantly correlated with HbA1c values 1–2 months before and 1 month after CGM. The nocturnal blood glucose levels were significantly correlated with HbA1c values 1–3 months before and 1–2 months after CGM.

**Conclusions:**

CGM can predict HbA1c values within 1 month after CGM in patients with DM.

## Introduction

Clinical investigations have illustrated the correlation of hemoglobin A1c (HbA1c) values with both microvascular and macrovascular complications in patients with diabetes mellitus (DM) [1, 2]. Strict glycemic treatment plays a crucial role in preventing the development and progression of long-term complications associated with DM. HbA1c values are associated with blood glucose levels over the lifetime of red blood cells (approximately 120 days) and are the current gold standard for clinical monitoring of glycemic control in DM [3, 4]. A study reported a strong correlation between HbA1c values and mean blood glucose levels by using the 7-point blood glucose profiles [5]. Accordingly, HbA1c values were hypothesized to represent relatively long-term glycemic status in patients with DM [6]. The International Diabetes Federation and American Diabetes Association have reported that the HbA1c value < 7.0% is a target for improving DM control [7, 8]. However, intensive treatment of DM is accompanied by hypoglycemia. Severe hypoglycemia may be a critical cause of morbidity. Furthermore, the optimal strategy for monitoring HbA1c values in patients with DM is not well established.

Glucose monitoring is a crucial aspect of DM control. The current accessibility of continuous glucose monitoring (CGM) systems is considered a valuable development in the management of DM. CGM enables the recording of various glucose data, including glucose excursions, patterns, and trends, and timepoints of the associated changes, in an attempt to optimize glycemic control [9, 10]. Recent meta-analyses have revealed that CGM system-based blood glucose monitoring is more effective for glycemic control in patients of type 1 and 2 DM than self-monitoring of blood glucose [11–13]. However, approved CGM systems demonstrate suboptimal accuracy [14–16]. In this study, we investigated the relationship between CGM system results and HbA1c values and evaluated which time points of HbA1c values were related to CGM as well as CGM parameters correlated with glycemic control in patients with DM.

## Materials and methods

### Study population

We retrospectively analyzed data of patients with DM who received CGM. CGM was performed using iPro2 (Medtronic, Northridge, CA, USA) for 5 days and 24-h CGM data were extracted from the first or second day of glucose monitoring. In total, 91 patients with DM received CGM, of which 27 (30%) had type 1 DM and 64 (70%) had type 2 DM. The age, body height, and weight, comorbidity, and history of medications of each participant were recorded. HbA1c values were measured using Sebia Capillarys 2 Flex Piercing (Sebia Electrophoresis, France). This capillary electrophoresis-based HbA1c assay had good analytical performances and a high correlation to other high-performing assays [17]. The HbA1c data were collected from 3 months before and 3 months after CGM. Very high (> 400 mg/dL) and very low (< 40 mg/dL) blood glucose levels were recorded through CGM, and patients with anemia were excluded. The blood glucose levels recorded from 0600 to 2200 represented diurnal blood glucose, and those from 2200 to 0600 represented nocturnal blood glucose levels. The whole-day, diurnal, and nocturnal glucose levels during CGM were calculated based on the average of the highest 10% (CGM_H10%_), 25% (CGM_H25%_), and 50% (CGM_H50%_) or all data (CGM_100%_). The mean amplitude of glycemic excursions (MAGE), classical standard deviation (SD), continuous overlapping net glycemic action (CONGA), and calculated coefficient of variation (CV) were calculated using Glycemic Variability Analyzer Program 1.1 (MATLAB 2010b; MathWorks, USA).

### Statistical analysis

Continuous variables are expressed as mean ± SD and were compared using the Student t-test. Categorical variables were reported as frequencies and compared using the χ^2^ or Fisher exact test if at least one cell had an expected cell count of < 5. A two-tailed P < 0.05 was considered statistically significant. A linear correlation was used to correlate the measured parameters. All statistical analyses were performed on SigmaPlot (version 12.3). The correlations among the HbA1c at different time points and were compared using the cocor package (https://comparingcorrelations.org/) [18].

## Results

Of the 91 patients with DM who received CGM, 9 patients with markedly elevated or very low blood glucose levels were excluded; of the remaining patients, 23 and 59 were diagnosed as having type 1 and 2 DM, respectively (Table 1). Patients with type 1 DM were younger and taller than patients with type 2 DM. The duration of DM was 15.3 ± 8.8 years. The mean C-peptide level among the 23 patients with type 1 DM was 0.2 ± 0.14 ng/mL. However, only 27 patients with type 2 DM have C-peptide data, and the mean level was 2.39 ± 2.08 ng/mL. Among these 82 DM patients, 23 had diabetic retinopathy, 22 had diabetic nephropathy, and 30 had diabetic neuropathy. Of these 82 patients, 67 patients adjusted their treatment during the study period. Forty patients increased the dosage of insulin, 17 decreased the dosage of insulin, 6 increased non-insulin anti-diabetic drugs, and 4 decreased non-insulin anti-diabetic drugs. Among the 82 DM patients enrolled in our study, 74 patients have checked HbA1c levels more than twice, and these HbA1c levels were evaluated in correlation with the CGM at different time points. Table 2 illustrates that the blood glucose levels (CGM_100%_) measured through CGM were significantly correlated with HbA1c values measured 1 (r = 0.69) and 2 (r = 0.39) months after CGM and 1–3 months (r = 0.35) before CGM. The HbA1c values 1 month after CGM was significantly correlated with the highest 10% (CGM_H10%_, r = 0.72), 25% (CGM_H25%_, r = 0.74), and 50% (CGM_H50%_, r = 0.73) measured from CGM. The CGM_H50%_ of blood glucose levels was significant with HbA1c values 3 months before and 1 month after CGM, but not with HbA1c values 1–2 months before and 2–3 months after CGM. However, the CGM_H10%_ and CGM_H25%_ of blood glucose levels during CGM did not correspond to HbA1c values measured 13 months before and 2–3 months after CGM. The diurnal blood glucose levels were significantly correlated with HbA1c values 1 and 2 months before and 1 month after CGM. The nocturnal blood glucose levels were significantly correlated with HbA1c values 1–3 months before and 1–2 months after CGM.

**Table 1:** Baseline characteristics of study participants

	All (n = 82)	Type 1 DM (n = 23)	Type 2 DM (n = 59)
Age (year)	59.7 ± 16.2	46.2 ± 17.1*	64.9 ± 12.4
Sex (% women)	44 (54)	11 (48)	33 (56)
Body weight (kg)	65.5 ± 12.3	66.6 ± 15.1	65.1 ± 11.1
Body height (m)	1.62 ± 0.09	1.65 ± 0.07*	1.60 ± 0.09
BMI (kg/m^2^)	25 ± 4	24 ± 4	25 ± 3
Duration of DM (year)	15.3 ± 8.8	15.3 ± 10.3	15.3 ± 8.3
Mean glucose level (mg/dL)	156.6 ± 36.6	152.2 ± 27.1	158.3 ± 40.1
Complication of DM			
Retinopathy (%)	23 (28%)	4 (17%)	19 (32%)
Nephropathy (%)	22 (27%)	4 (17%)	18 (31%)
Neuropathy (%)	30 (37%)	5 (22%)	25 (42%)
Insulin (%)	71 (87%)	23 (100%)*	48 (81%)
Sulfonylurea (%)	8 (10%)	0 (0%)	8 (14%)
Glinides (%)	2 (2%)	0 (0%)	2 (3%)
Metformin (%)	37 (45%)	1 (4%)*	36 (61%)
α-Glucosidase inhibitor (%)	11 (13%)	0 (0%)*	11 (19%)
Thiazolidinedione (%)	4 (5%)	0 (0%)	4 (7%)
DPP-4 inhibitor (%)	23 (28%)	0 (0%)*	23 (39%)
SGLT2i (%)	6 (7%)	0 (0%)	6 (10%)
GLP-1 RA (%)	1 (1%)	0 (0%)	1 (2%)
Change of treatment			
Increase insulin dose (%)	40 (49%)	14 (61%)	26 (44%)
Decrease insulin dose (%)	17 (21%)	4 (17%)	13 (22%)
Increase oral antidiabetic agent (%)	6 (7%)	0 (0%)	6 (10%)
Decrease oral antidiabetic agent (%)	4 (5%)	0 (0%)	4 (7%)
SD (mg/dL)	44.3 ± 16.6	55.1 ± 18.1*	39.6 ± 13.6
MAGE (mg/dL)	117.7 ± 34.0	135.4 ± 31.8*	110.2 ± 32.3
CONGA-1 (mg/dL)	29.9 ± 9.1	35.4 ± 8.1*	27.6 ± 8.5
%CV	28.5 ± 9.6	35.2 ± 10.2*	25.6 ± 7.7

DM: diabetes mellitus; BMI: body mass index; HbA1c: hemoglobin A1c; DPP-4: dipeptidyl peptidase 4; SGLT2i: sodium-glucose co-transporter 2 inhibitors; GLP-1 RA: glucagon-like peptide-1 receptor agonist; SD: standard deviation; MAGE: mean amplitude of glucose excursion; CONGA: Continuous overlapping net glycemic action (1 h intervals); %CV: percentage coefficient of variation; Data are mean ± SD

*Means type 1 DM versus type 2 DM; P < 0.05

**Table 2:** Correlation of HbA1c with mean continuous glucose level

	After 1 month (n = 25)	After 2 month (n = 34)	After 3 month (n = 36)	Before 1 month (n = 55)	Before 2 month (n = 31)	Before 3 month (n = 36)
Total glucose	0.69*	0.39*	0.16^#^	0.35*	0.26^#^	0.42*
Highest 10% glucose	0.72*	0.13^#^	0.19^#^	0.22^#^	0.18^#^	0.31^#^
Highest 25% glucose	0.74*	0.16^#^	0.20^#^	0.25^#^	0.14^#^	0.33^#^
Highest 50% glucose	0.73*	0.25^#^	0.19^#^	0.28*^#^	0.16^#^	0.37*
Diurnal glucose	0.73*	0.28^#^	0.08^#^	0.28*^#^	0.36*	0.30^#^
Nocturnal glucose	0.53*	0.49*	0.21	0.35*	0.04	0.45*

HbA1c: hemoglobin A1c

*Means P < 0.05; ^#^means P < 0.05 compared with r value of the group after 1 month

Table 3 illustrates that the blood glucose levels (CGM_100%_) measured through CGM were significantly correlated with HbA1c values measured 1 (r = 0.71) and 2 (r = 0.44) months after CGM and 1 month (r = 0.35) before CGM in the treatment-adjusted group. The HbA1c values 1 month after CGM was significantly correlated with the highest 10% (CGM_H10%_, r = 0.70), 25% (CGM_H25%_, r = 0.73), and 50% (CGM_H50%_, r = 0.73) measured from CGM. The diurnal blood glucose levels were significantly correlated with HbA1c values 1 month before and 1 month after CGM. The nocturnal blood glucose levels were significantly correlated with HbA1c values 1–3 months before and 1–2 months after CGM.

**Table 3:** Correlation of HbA1c with mean continuous glucose level in treatment-adjusted group

	After 1 month (n = 23)	After 2 month (n = 28)	After 3 month (n = 28)	Before 1 month (n = 46)	Before 2 month (n = 25)	Before 3 month (n = 29)
Total glucose	0.71*	0.44*	0.09^#^	0.35*	0.33	0.33
Highest 10% glucose	0.70*	0.22^#^	0.05^#^	0.30*^#^	0.30	0.23^#^
Highest 25% glucose	0.73*	0.26^#^	0.09^#^	0.29*#	0.26^#^	0.25^#^
Highest 50% glucose	0.73*	0.32^#^	0.10^#^	0.29*^#^	0.27^#^	0.27^#^
Diurnal glucose	0.74*	0.32^#^	0.04^#^	0.29*^#^	0.40	0.19^#^
Nocturnal glucose	0.55*	0.53*	0.11	0.31*	0.08	0.40*

HbA1c: hemoglobin A1c

*Means P < 0.05; ^#^means P < 0.05 compared with r value of the group after 1 month

Table 4 displays the glycemic variation of patients with type 1 and 2 DM over 3 months before and 3 months after CGM. The mean, SD, MAGE, CONGA, and CV values were calculated for each patient. Table 5 displays the relationship between HbA1c and glycemic variation, including SD, percent CV (%CV), MAGE, and CONGA. Overall, the SD and MAGE were significantly correlated to HbA1c values in the next 1 month, and the %CV was significantly correlated to HbA1c values 2 months after CGM. The %CV was significantly correlated with the HbA1c values 2 months after CGM in patients with type 1 DM. In patients with type 2 DM, the SD and MAGE were significantly correlated to HbA1c values 1 month after CGM and the %CV was significantly correlated with HbA1c values 2 months after CGM.

**Table 4:** HbA1c and glycemic variations in type 1 and type 2 DM patient

	Total DM patient					
	After 1 month (n = 25)	After 2 month (n = 34)	After 3 month (n = 36)	Before 1 month (n = 55)	Before 2 month (n = 31)	Before 3 month (n = 36)
HbA1c	8.1 ± 1.4	8.4 ± 1.4	7.6 ± 1.2	8.5 ± 1.2	8.2 ± 1.2	8.2 ± 1.5
Mean glucose level (mg/dL)	158.1 ± 45.8	156.6 ± 36.8	155.7 ± 28.2	158.0 ± 30.5	170.5 ± 43.3	151.6 ± 30.4
SD (mg/dL)	39.1 ± 11.1	43.9 ± 17.7	45.1 ± 15.7	43.1 ± 13.4	45.8 ± 19.4	43.9 ± 13.6
MAGE (mg/dL)	108.1 ± 23.7	119.0 ± 35.5	117.0 ± 26.6	118.8 ± 33.8	120.0 ± 34.3	113.2 ± 30.0
CONGA-1 (mg/dL)	27.0 ± 7.5	30.6 ± 10.1	30.0 ± 7.8	29.8 ± 8.5	29.8 ± 8.7	29.9 ± 8.2
%CV	25.5 ± 7.4	28.4 ± 10.6	29.2 ± 9.5	27.6 ± 8.5	27.4 ± 10.9	29.3 ± 8.8

	Type 1 DM patient					
	After 1 month (n = 7)	After 2 month (n = 9)	After 3 month (n = 11)	Before 1 month (n = 13)	Before 2 month (n = 10)	Before 3 month (n = 10)
HbA1c	7.7 ± 1.0	8.6 ± 1.2	8.1 ± 1.2	8.5 ± 1.1	8.0 ± 1.5	9.0 ± 1.8
Mean glucose level (mg/dL)	141.7 ± 19.0	147.7 ± 26.1	158.8 ± 24.8	158.2 ± 23.5	154.0 ± 31.0	150.0 ± 27.3
SD (mg/dL)	41.9 ± 10.0	56.4 ± 17.9	56.6 ± 16.4	55.0 ± 11.2	57.5 ± 25.4	49.5 ± 12.7
MAGE (mg/dL)	111.4 ± 17.4	138.6 ± 30.6	127.6 ± 16.9	140.2 ± 26.3	136.4 ± 39.5	122.2 ± 23.1
CONGA-1 (mg/dL)	31.8 ± 6.2	37.8 ± 7.6	34.9 ± 6.5	37.5 ± 7.2	35.7 ± 9.7	33.3 ± 8.0
%CV	29.5 ± 5.1	38.3 ± 9.5	34.0 ± 10.9	33.6 ± 9.6	36.6 ± 11.5	31.4 ± 10.5

	Type 2 DM patient					
	After 1 month (n = 18)	After 2 month (n = 25)	After 3 month (n = 25)	Before 1 month (n = 42)	Before 2 month (n = 21)	Before 3 month (n = 26)
HbA1c	8.3 ± 1.5	8.4 ± 1.5	7.4 ± 1.2	8.4 ± 1.2	8.2 ± 1.0	7.9 ± 1.2
Mean glucose level (mg/dL)	164.5 ± 51.8	159.4 ± 40.0	153.0 ± 31.9	158.7 ± 33.1	173.8 ± 49.3	156.5 ± 32.5
SD (mg/dL)	37.7 ± 11.9	39.6 ± 15.9	40.7 ± 13.7	39.7 ± 12.6	39.8 ± 49.3	42.9 ± 14.3
MAGE (mg/dL)	109.1 ± 25.2	114.6 ± 33.9	109.6 ± 28.7	112.7 ± 33.9	115.2 ± 27.7	109.1 ± 33.7
CONGA–1 (mg/dL)	25.4 ± 7.2	28.0 ± 9.56	28.3 ± 8.5	28.0 ± 8.2	27.2 ± 6.6	29.2 ± 9.0
%CV	24.0 ± 7.7	24.8 ± 8.5	27.2 ± 8.2	25.7 ± 7.3	23.1 ± 7.4	28.5 ± 8.1

HbA1c: hemoglobin A1c; SD: standard deviation; %CV: percentage coefficient of variation; MAGE: mean amplitude of glucose excursion; CONGA: Continuous overlapping net glycemic action (1 h intervals)

**Table 5:** Correlation between HbA1c and glycemic variations in type 1 and type 2 DM patient

	Total DM patient					
	After 1 month (n = 25)	After 2 month (n = 34)	After 3 month (n = 36)	Before 1 month (n = 55)	Before 2 month (n = 31)	Before 3 month (n = 36)
Mean glucose level (mg/dL)	0.69*	0.39*	0.16^#^	0.35*	0.26^#^	0.42*
SD (mg/dL)	0.49*	0.20	0.17	0.01^#^	0.15	0.06
MAGE (mg/dL)	0.46*	0.06	0.30	0.18	0.17	0.17
CONGA-1 (mg/dL)	0.30	0.20	0.21	0.03	0.122	0.002
%CV	0.03	0.37*	0.15	0.22	0.25	0.16

	Type 1 DM patient					
	After 1 month (n = 7)	After 2 month (n = 9)	After 3 month (n = 11)	Before 1 month (n = 13)	Before 2 month (n = 10)	Before 3 month (n = 10)
Mean glucose level (mg/dL)	0.56	0.12	0.26	0.07	0.16	0.07
SD (mg/dL)	0.59	0.52	0.15	0.48	0.32	0.35
MAGE (mg/dL)	0.65	0.14	0.14	0.09	0.01	0.12
CONGA-1 (mg/dL)	0.36	0.32	0.19	0.002	0.22	0.43
%CV	0.39	0.71*	0.09	0.19	0.30	0.14

	Type 2 DM patient					
	After 1 month (n = 18)	After 2 month (n = 25)	After 3 month (n = 25)	Before 1 month (n = 42)	Before 2 month (n = 21)	Before 3 month (n = 26)
Mean glucose level (mg/dL)	0.70*	0.45*	0.08^#^	0.32*	0.38	0.57*
SD (mg/dL)	0.52*	0.18	0.18	0.07	0.16	0.11
MAGE (mg/dL)	0.47*	0.06	0.17	0.26	0.27	0.25
CONGA-1 (mg/dL)	0.44	0.24	0.09	0.01	0.007	0.05
%CV	0.02	0.45*	0.06	0.27	0.18	0.27

HbA1c: hemoglobin A1c; SD: standard deviation; %CV: percentage coefficient of variation; MAGE: mean amplitude of glucose excursion; CONGA: Continuous overlapping net glycemic action (1 h intervals)

*Means P < 0.05; ^#^means P < 0.05 compared with r value of the group after 1 month

## Discussion

The goal of DM treatment is to prevent chronic complications [19, 20]. Glucose monitoring is crucial in DM control. HbA1c assay is recommended as the optimal approach to monitoring DM glycemic control [21] and is used globally as the basis of adjustment in treatment guidelines [22–24]. A study reported a strong correlation between HbA1c values and mean blood glucose levels using 7-point blood glucose profiles [5]. However, the optimal strategy for monitoring HbA1c values was not clear because the relationship between DM control and HbA1c values in intensive glucose monitoring is not well established. CGM could provide more blood glucose data and contribute to the improvement in DM management, as demonstrated by the significant lowering of HbA1c values in patients with DM [25–28]. However, studies have noted strong correlations between CGM interstitial glucose and venous plasma or capillary glucose levels [29–31]. Studies have reported a less than the acceptable correlation between CGM interstitial glucose results and venous plasma or capillary glucose levels [32, 33]. In this study, we determined that CGM may correlate well with HbA1c values over 3 months before and 3 months after CGM. The CGM was most closely related to HbA1c values in the next month after CGM when measured based on the interstitial glucose levels every 5 min for 5 days. This phenomenon was also observed with patients who have an adjustment of treatment, which means that CGM quickly corresponds to the adjustment of treatment, and seems to be closely associated with the HbA1c values one month after CGM.

Sharp and Rainbow have demonstrated that CGM was strongly correlated with HbA1c values at the time of insertion [34]. Saladri et al. also identified a correlation between the area under the glucose curve of CGM and HbA1c values in patients with type 1 DM [35]. Furthermore, Nathan et al. extensively analyzed CGM and identified a strong correlation between the mean glucose measured by CGM and HbA1c values [31]. Glucose variability may be a factor in DM complications [36]. An acute increase in blood glucose can produce significant alterations in normal homeostasis, leading to endothelial dysfunction and inflammation, among other effects [37]. A study indicated that MAGE was not correlated to HbA1c values [38]. In this study, we determined that MAGE was poorly associated with HbA1c values at several time points but was significantly correlated with the HbA1c values 1 month after CGM, particularly in patients with type 2 DM. Furthermore, CGM is vital in improving glucose variability to achieve strict glycemic control.

Several factors may alter the relationship between the mean blood glucose levels and HbA1c values. For instance, variable red cell turnover may be affected by hyperglycemia [39]. Moreover, glycation rates may differ among individuals at the same mean blood glucose levels [40]. Notably, the negative correlation between measured mean glucose levels and HbA1c values 3 months after CGM may also suggest an improvement in the mean glucose levels after the intervention based on the CGM results.

In conclusion, the association of CGM results with HbA1c values 1 month after monitoring was positive. Furthermore, CGM could record glucose variability and is a reliable tool to assess glycemic state and improve DM management.

## Limitations

The limitations of this study should be considered. First, the r value in our study is relatively low compared with previous studies [18, 41]. This difference may be due to the small sample in each group in this single-center study. Second, HbA1c reflects the average blood glucose of 3 months, and the collected dynamic blood glucose data is the blood glucose data of a certain day. A larger sample size will be needed to further explore the relationship between the two. Third, the CGM data were retrospectively collected from the medical history, and HbA1c data were not integrated.

## Data Availability

The data used to support the findings of this study are available from the corresponding authors upon request.
